# Case report: The CCDC103 variant causes ultrastructural sperm axonemal defects and total sperm immotility in a professional athlete without primary ciliary diskinesia

**DOI:** 10.3389/fgene.2023.1062326

**Published:** 2023-01-26

**Authors:** Francesca Paola Luongo, Alice Luddi, Rosetta Ponchia, Rossella Ferrante, Sara Di Rado, Eugenio Paccagnini, Mariangela Gentile, Pietro Lupetti, Raffaella Guazzo, Alfredo Orrico, Liborio Stuppia, Paola Piomboni

**Affiliations:** ^1^ Department of Molecular and Developmental Medicine, University of Siena, Siena, Italy; ^2^ Department of Psychological Sciences, Health and Territory, University of Chieti-Pescara, Chieti, Italy; ^3^ Department of Life Sciences, University of Siena, Siena, Italy; ^4^ Pathology Unit, Siena University Hospital, Siena, Italy; ^5^ Assisted Reproductive Unit, Siena University Hospital, Siena, Italy

**Keywords:** sperm immotility, axoneme, ccdc103, nasal cilia, dynein arms, infertility

## Abstract

Primary ciliary dyskinesia (PCD) is an inherited autosomal-recessive disorder characterized by abnormal ciliary motion, due to a defect in ciliary structure and/or function. This genetic condition leads to recurrent upper and lower respiratory infections, bronchiectasis, laterality defect, and subfertility. Male infertility is often associated with PCD, since the ultrastructure of the axoneme in the sperm tail is similar to that of the motile cilia of respiratory cells. We present the first reported case of a male patient from a non-consanguineous Italian family who exhibited a severe form of asthenozoospermia factor infertility but no situs inversus and absolutely no signs of the clinical respiratory phenotype, the proband being a professional basketball player. Whole-exome sequencing (WES) has identified a homozygote mutation (CCDC103 c.461 A>C, p.His154Pro) in the proband, while his brother was a heterozygous carrier for this mutation. Morphological and ultrastructural analyses of the axoneme in the sperm flagellum demonstrated the complete loss of both the inner and outer dynein arms (IDA and ODA, respectively). Moreover, immunofluorescence of DNAH1, which is used to check the assembly of IDA, and DNAH5, which labels ODA, demonstrated that these complexes are absent along the full length of the flagella in the spermatozoa from the proband, which was consistent with the IDA and ODA defects observed. Noteworthy, TEM analysis of the axoneme from respiratory cilia showed that dynein arms, although either IDAs and/or ODAs seldom missing on some doublets, are still partly present in each observed section. This case reports the total sperm immotility associated with the CCDC103 p.His154Pro mutation in a man with a normal respiratory phenotype and enriches the variant spectrum of ccdc103 variants and the associated clinical phenotypes in PCD, thus improving counseling of patients about their fertility and possible targeted treatments.

## Introduction

The movement of sperm cells is due to the axonemal complex, where the motile activity is generated by two series of adenosine triphosphate (ATP)-dependent dynein arms, that generates a sliding force that is transformed into a wave throughout the whole sperm tail by accessory cytoskeletal structures. According to the number of heavy-chain motors within each dynein complex, different group of dyneins may be distinguished; usually, heavy chains are considerable proteins composed of about 4500 residues. Apart from dyneins, additional components are required for the assembly of the final motor complexes as well as for the regulation of their movement. Indeed, the axoneme assembly is a very complex process, and the mechanism of the correct arrangement of all the dynein arms on the doublet microtubules is still a key question (King, 2016). As regard to the outer arms to interact with the doublet microtubules, several docking complexes including coiled-coil proteins (DC1 and DC2) and a calmodulin homolog (DC3) are needed (Takada and Kamiya, 1994). More recently, an additional protein, namely, coiled-coil domain containing-103 (ccdc103), has been demonstrated to be essential for outer arm insertion into the microtubule structure (texture) (Panizzi et al., 2012). ccdc103 has very important biophysical properties (e.g., a very high melting temperature) as well as the ability to form highly stable dimers and/or oligomers. Due to these peculiar properties, it has been supposed that ccdc103 polymerization during axonemal growth may provide, along the outer doublets, a high-affinity road with which the heterotrimeric outer arms/docking complex (ODA/DC) can then associate (Takada and Kamiya, 1994; Owa et al., 2014). Mutations in ccdc103 have been detected in primary ciliary dyskinesia (PCD, MIM 244400) patients with situs inversus totalis and absence of dynein arms in the sperm (Shoemark et al., 2018; Pereira et al., 2019). PCD, whose estimated global prevalence is about 1:10000 live-born children (Afzelius and Stenram, 2006; Hannah et al., 2022), is a heterogeneous autosomal recessive disease characterized by abnormal ciliary motion, due to a defect in ciliary structure and/or function. This genetic condition leads to recurrent upper and lower respiratory tract infections, bronchiectasis, laterality defect, and subfertility (Mirra et al., 2017). Male infertility is often associated with PCD, since the ultrastructure of the axoneme in the sperm tail is similar to that of motile cilia of respiratory cells (Sironen et al., 2020). Indeed, numerous mutations in more than 40 genes have been so far identified causing asthenozoospermia in PCD patients (Olm et al., 2015; Boaretto et al., 2016). Motility defects, indeed, have been identified in men bearing mutations in CFAP300 and in DNAF2 that show complete absence of dynein arms; the lack of inner dynein arms is a feature of sperm bearing mutations in DNAH1 and DNAH2, while mutations of CCDC40 induce axonemal disorganization and complete asthenozoospermia. Likewise, mutations of proteins forming the radial spokes (CFAP251) and the central pairs (e.g., AK7 and ARMC2) cause total sperm immotility (Sironen et al., 2020).

A high heterogeneity has been reported associated to the missense mutation p.His154pro (rs145457535) in ccdc103, with patients showing different degrees of axonemal defects, ranging from normal ultrastructure to partial or complete ODA/IDA defects (Casey et al., 2015; Shoemark et al., 2018).

The availability of multiple algorithms for diagnosis (Shoemark et al., 2019) and the presence of PCD phenotype heterogeneity still make the PCD confirmation a challenge. Therefore, a better knowledge of the pathogenic effect of mutation in PCD-related genes is advised.

## Patients and methods

The patient, a 40-year-old man from a non-consanguineous family, underwent spermatological and genetic analyses during an infertility evaluation for the search of a pregnancy at the ART Centre of the Siena University Hospital, Italy. The most recurrent causes for male infertility (including hormone levels, chromosomal defects, and Y-chromosome microdeletion) were excluded. The medical history of the subject was recovered, and respiratory complaints were excluded. Moreover, chest X-ray revealed normal left/right body symmetry. Ejaculated sperm, nasal cilia, and peripheral blood samples were collected from the patient; peripheral blood sample was also collected from the patient’s brother. All the participants provided written informed consent for the use of their samples and data. This study was approved by the University of Siena’s ethics committee (CEAVSE, protocol number 18370, 2/10/2020). All experiments were performed in accordance with the relevant guidelines and regulations.

### Genomic DNA preparation and whole-exome sequencing

Exome sequencing was performed on the patient by extracting DNA from the blood and ejaculated sperm using QIAamp DNA Blood Kits (Qiagen, Hilden, Germany) following the manufacturer’s instructions. Whole-exome sequencing (WES) was performed using the Ion Torrent S5 system (Thermofisher, Applied Biosystem; Foster City, CA, United States) after library preparation using Ion AmpliSeq™ Exome RDY Kit 4 × 2 (Thermofisher, Applied Biosystem; Foster City, CA, United States), consisting of fragmentation and adapter ligation onto the PCR products and clonal amplification. The sequencing step was carried out with Ion Torrent specific equipment and reagents according to the protocols. The prepared samples of ion sphere particles (ISPs) were loaded onto an Ion 550™ chip with the Ion Chef (Thermofisher, Applied Biosystem; Foster City, CA, United States), and sequencing was performed using the Ion S5™ sequencing reagents (Thermofisher, Applied Biosystem; Foster City, CA, United States). NGS data analysis was performed using Ion Reporter 5.14 software (Thermofisher; Foster City, CA, United States), which performs alignment between the obtained data and the Hg19 Human reference genome. Specifically, 34 genes related to PCD/Kartagener syndrome were selected and analyzed (gene table, Supplementary File S3). The sequencing analysis produced a total of 293,903 amplicons. The uniformity of base coverage was over 98% in all batches, and the base coverage was over 20 X at all target regions. Sanger sequencing was used for the confirmation of the gene variant detected by WES.

### Transmission electron microscopy

Samples of nasal mucosa and ejaculated sperm were fixed in 2.5% glutaraldehyde diluted in 0.1 M sodium cacodylate buffer, pH 7.2 for 1 h, washed in cacodylate buffer overnight, and postfixed in 1% osmium tetroxide for 1 h at 4°C. The samples were dehydrated with a graded series of ethanol and embedded in epoxy resin. In order to improve microtubule’s visualization, a rate of ejaculated sperm was fixed in a mixture of 2% glutaraldehyde and 1% tannic acid in 0.1 M phosphate buffer. The sample was left in the fixative for a week and then washed 10 times for 5 min in distilled water; after that, the sample was stained for 2 h in 1% uranyl acetate in distilled water (Afzelius et al., 1995). The sample was dehydrated and embedded as previously described. Ultrathin sections of 70 nm were collected on copper grids, stained with uranyl acetate and lead citrate, and observed with an FEI Tecnai G2 Spirit transmission electron microscope (Hillsboro, OR, United States) equipped with a TVIPS CMOS camera TemCam F-216 (Gauting, Germany).

### Immunofluorescence analysis

Immunofluorescence on the spermatozoa was performed after fixation with 4% PFA for 25 min as previously described (Luddi et al., 2022). Once resuspended, the samples were smeared into glass slides and left to dry. The slides were incubated in a blocking solution containing 5% goat normal serum in 1% PBS/BSA (bovine serum albumin) and then incubated overnight at 4°C with DNAH1, DNAH5, and anti-β-tubulin primary antibodies (RRID: AB_10670849, AB_10672791, and AB_1844090, respectively) used according to the manufacturer’s instructions. The slides were washed three times in PBS and incubated with FITC-labeled anti-rabbit and TRITC-labeled anti-mouse secondary antibodies (Supplementary Table S1). After washing in PBS, the slides were stained with 4′,6-diamidino-2-phenylindole (DAPI) (LifeTechnologies, Carlsbad, CA, United States) to counterstain the nuclei, mounted with DABCO, and then, observed with a Leica DM6000 microscope (Leica Mycrosistem, Wetzlar, Germany). Images were captured with a CFTR6500 digital camera (Leica Mycrosistem, Wetzlar, Germany). The specificity of immunostaining was confirmed by using pre-immune sera instead of the primary antibody, followed by incubation with the secondary antibody.

### Bioinformatics

The PubMed (https://pubmed.ncbi.nlm.nih.gov/), OMIM (https://secure.jhu.edu/form/OMIM), HGMD (https://www.hgmd.cf.ac.uk/ac/index.php), ClinVar (https://www.ncbi.nlm.nih.gov/clinvar/), and gnomAD v2.1.1 (https://gnomad.broadinstitute.org/) databases and the professional version of the Human Splicing Finder system (HSF_Pro) were used to evaluate the pathogenicity of the variants. Variant annotation was carried out through TVC- Torrent Variant Caller and Ion Reporter 5.14. The mutated protein was predicted by MutPred2 and Polyphen2 and analyzed by DynaMut software (Rodrigues et al., 2018). CCDC103 protein Q8IW40.pdb and the point amino acid mutation H154P were analyzed by DynaMut software. The impact of amino acid changes in any given protein is predicted based on the positive or negative changes in Gibbs free energy (ΔΔG°) between unfolded *versus* folded states in that protein. It is well known that a protein ΔΔG° can represent its stability as a value. STRING analysis was performed by using STRING network building tool software (https://string-db.org) by the “multiple protein” option. *Homo sapiens* was selected as the preferred “organism.” In the “settings” section, “molecular action” was selected as the preferred interaction among proteins to be visualized. Textmining, experiments, databases, and co-expression were selected as active interaction sources. The minimum required interaction score was 0.700, which was considered high confident. No more than five interactors were permitted to be added. The network view summarizes the predicted associations for a particular group of proteins. The nodes represent proteins, while the edges represent the predicted functional associations.

## Results

### Semen analysis and ultrastructural evaluation of the axoneme structure

**FIGURE 1:**
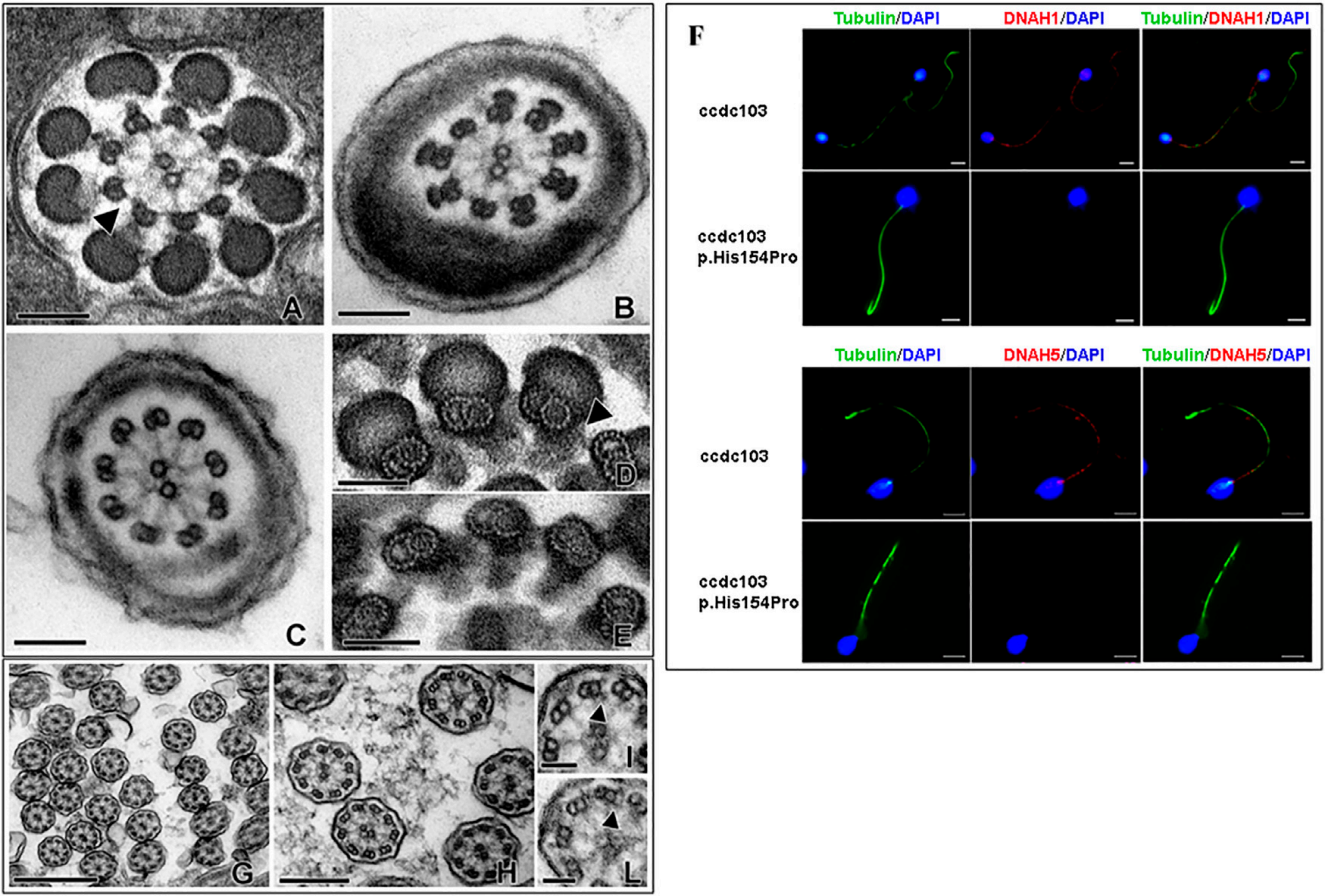
Electron microscopy micrographs of different section levels of the spermatozoa flagellum with a focus on IDA and ODA **(A–C)**, spermatozoa samples treated with tannic acid **(D, E),** and nasal mucosa samples **(G–L)**. Scale bar for A-B-C 100 nm, D-E-H-I 50 nm, F 500 nm, and G 200 nm. Immunofluorescence **(F)** of the ejaculated sperm revealed the absence of both DNAH1 and DNAH5 in the patient (red) compared to the normal control. An anti-tubulin antibody was used to mark the sperm axoneme (green), and DAPI was used to label the nuclei (blue).

The patient, a 40-year-old man from a non-consanguineous family, underwent spermatologic and genetic analyses during an infertility evaluation for the search of a pregnancy at the ART Centre of the University Hospital, at Siena, Italy. The most recurrent causes for male infertility (including hormone levels, chromosomal defects, and Y-chromosome microdeletions) were excluded, and semen analyses at light microscopy according to the WHO (WHO laboratory manual for the examination and processing of human semen, 2022) were repeated four times during a period of 2 years showing sperm parameters over the 5^th^ percentile, except for the progressive motility, which resulted in all sample being completely absent (0%) (Supplementary Table S2). To further characterize the gamete ultrastructure, we carried out transmission electron microscopy (TEM) of the ejaculated sperm. TEM of sperm tails sections conducted at different sectioning levels revealed the complete absence in all the examined cells of both the inner and outer dynein arms (IDA and ODA, respectively), the microtubule-associated molecular motors with ATPase activity which, by promoting the reciprocal sliding of doublets, enable flagellar beating (Figures 1A–C). The same scenario was also confirmed in a rate of ejaculated sperm treated with tannic acid. In particular, in this sample, it was possible to evidence that the doublets have a canonical A- and B-tubule protofilament pattern (Figures 1D, E). No structural alterations of other axonemal components, such as nexin bridges, connecting adjacent microtubular doublet or radial spokes were identified.

In order to confirm the absence of the dynein arm protein along the sperm flagellum, we carried out immunofluorescence of DNAH1 (Figure 1F, upper panel), which is used to check the assembly of IDA and DNAH5 (Figure 1F, lower panel), which labels ODA, in sperm from the proband and unmutated individuals. In the normal spermatozoa, immunostaining of the proteins was located along the whole sperm tail, whereas DNAH1 and DNAH5 immunostaining was absent along the full length of the flagella in the sperm from the proband, which was consistent with the IDA and ODA absence observed at the TEM level (Figures 1A–C); nor the proteins were delocalized in other cellular compartments, such as cytoplasmic residues. The defect affecting the sperm axoneme is, therefore, compatible with PCD, which is clinically considered a severe risk factor for infertility. In order to confirm the diagnosis of PCD, nasal cilia samples were evaluated at the ultrastructural level. Our observations revealed that the dynein arms were not uniformly distributed along microtubular doublets with either IDAs and/or ODAs seldom missing on some doublets in the observed sections. Other detectable modifications of cytoskeletal axonemal components were not found (Figures 1G–L). The ultrastructural feature of the nasal ciliary axoneme is coherent with the absence of respiratory problems in the patient, despite the total sperm immotility.

### Identification of a homozygous mutation p.His154Pro in CCDC103

**FIGURE 2:**
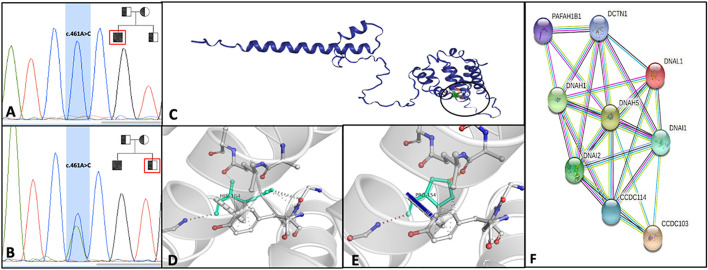
Electropherograms of the patients with ccdc103 c.461 A>C mutation in homozygous **(A)** and his heterozygote brother **(B)**; in the right corner of Images A and B, the family pedigree is shown. Squares and circles represent, respectively, males and females. Filled symbol represents the affected member, and slashed symbols represent deceased members. **(C)** DynaMut prediction of interactomic interaction of CCDC103. Wild-type **(D)** and mutant H154P **(E)** residues are colored in light green and represented as sticks alongside the surrounding residues involved in any interactions. **(F)** STRING analysis summarizing functional information about ccdc103 with respect to the biological process, gene ontology category and predicting possible associations among them. STRING networks consist of nodes (spheres) representing proteins, while edges represent the predicted mode of action among proteins.

We performed WES in the DNA extracted from the ejaculated sperm of the patient, and we found the homozygous variant c.461 A>C. This mutation results in the nucleotide substitution A>C in position 461 in exon 4 of the *CCDC103* gene and causes the substitution of the amino acid histidine with prolin in position 154 of the coded protein (p.His154Pro). Sanger sequencing confirmed the presence of this variant in the genomic DNA isolated from peripheral lymphocytes of the proband (Figure 2A), thus excluding a *de novo* germinal mutation. The genetic investigation of the brother, a fertile man without PCD symptoms, revealed the same variant in heterozygosis, definitively demonstrating their parents are obligate carriers of this mutation (Figure 2B).

This CCDC103 mutation has been previously reported with variable phenotypes (Casey et al., 2015; Pereira et al., 2019; Shoemark et al., 2019); to better understand its pathogenicity at the molecular level, we performed *in silico* analysis using DynaMut2 software. The prediction of the change in stability upon point mutations in proteins has many applications in protein analysis and engineering. The stability of the abovementioned single amino acid variants was analyzed by normal mode analysis (NMA) using DynaMut. The total predicted change in stability ΔΔG (kcal/mol) for CCDC103 amino acid point substitution p.His154Pro is .549 with a confidence estimation of .802. ΔΔGpred <.0 indicates a stabilizing estimation, confidence estimation given as a value between .0 (not reliable) and 1.0 (highly reliable) (Figures 2C–E).

The STRING analysis (http://string-db.org/) of the interactors of CCDC103 pointed to a number of related proteins, most notably, DNAH1, DNAH5, DNAI1, and DNAI2 (Figure 2F). Proteins reported in the net are involved in outer and inner dynein arms assembly and regulation of cilium movement biological processes. Moreover, among diseases–genes associations are highlighted *situs inversus* and PCD, and among GO, dynein intermediate chain binding (GO:0045505), ATP-dependent microtubule motor activity, minus-end-directed (GO:0008569), and dynein light intermediate chain binding (GO:0051010) were identified.

## Discussion

This study simultaneously evaluated the effect of the CCDC103 p.His154Pro gene mutation on the ultrastructure of the axoneme in both respiratory cilia and sperm flagellum. CCDC103 p.His154Pro was previously reported as a high prevalence mutation causing PCD (Casey et al., 2015; Shoemark et al., 2018; Zubair et al., 2022). Anyway, the men carrying this homozygote mutation showed a fully immotile sperm but no *situs inversus* and absolutely no signs of clinical respiratory problems, the proband being a professional basketball player.

CCDC103 is an oligomeric coiled-coil domain protein that specifically binds and stabilizes polymerized microtubules, thus playing a critical role in the dynein arms assembly (King and Patel-King, 2015). Several studies demonstrated that the p.His154Pro variant in CCDC103 affected indistinctly both the cilia and the sperm flagellum, therefore hypothesizing that this mutation may act in a shared pathway of dynein arms formation in both cell types. This case reports evidence of different degrees of ultrastructural abnormalities in the axoneme of respiratory cells, with only a partial ODA and/or IDA lacking in analyzed axonemes. At the same time, we report the complete absence of both IDA and ODA arms in all sperm flagella. This significant pleiotropic effect may justify the total absence of respiratory problems in the patient, despite the complete sperm immotility.

A previous study reported this mutation affecting the axoneme of both cilia and sperm, thus suggesting that this variant in CCDC103 acts in a shared pathway of dynein arms formation in both cell types (Zubair et al., 2022). Anyway, some differences are known in the pathways regulating the axoneme structure of cilia and sperm, with NOTCH signaling required to activate the centriole amplification genes in cilia development, whereas WNT signaling playing a critical role in sperm flagellum formation (Choksi et al., 2014).

Our data are also supported by a recently published study demonstrating the tissue specific activity of this CCDC103. Indeed, CCDC103 forms dimers and higher order oligomers whose sizes appear tissue-specific (Pereira et al., 2019). Moreover, CCDC103 is expressed at a very high level in the sperm, while its expression in nasal cells is very low (Pereira et al., 2019). These data further confirm that CCDC103 may have a special role in sperm function and corroborate our findings showing a different spectrum of alteration in nasal *versus* sperm axoneme, where all IDA and ODA were missing.

*In silico* analysis classified with high confidence scores as deleterious this variation; the much reduced frequency of p.His154Pro in population databases further supports a presumed pathogenic role of this sequence variant.

In conclusion, we report the total sperm immotility associated with the CCDC103 p.His154Pro mutation in a man with a normal respiratory phenotype. Major limitation of this study is that we cannot exclude the fact that this proband is an unusual outlier, displaying a complete IDA and ODA loss phenotype exclusively in the sperm. Anyway, this case report, enriching the variant spectrum of CCDC103 variants and the associated clinical phenotypes in PCD, will improve the counseling for infertile patients with severe sperm motility problems suggesting a revision of the fertility guidelines that must include an accurate ultrastructural analysis of the ejaculated sperm. Moreover, the identification of this mutation has directed the diagnostic process toward the CCDC103 gene sequencing also in his partner; this allows offering a correct genetic counseling about the inheritance of this trait to the offspring. Transmission electron microscopy represents the gold standard to diagnose these genetic-based sperm defects (Baccetti et al., 2001) and should be offered to this type of patients, given the pleiotropic expression of CCDC103 we demonstrated, in accordance with the scientific literature.

## Data Availability

The original contributions presented in the study are publicly available. This data can be found here: https://www.ncbi.nlm.nih.gov/sra/PRJNA924559.
