# Supplementary material for: Case report: The CCDC103 variant causes ultrastructural sperm axonemal defects and total sperm immotility in a professional athlete without primary ciliary diskinesia
Source: Front Genet. 2023 Jan 26;14:1062326. doi: 10.3389/fgene.2023.1062326 (PMC9908957; doi:10.3389/fgene.2023.1062326)
Supplement: Supplementary file 1 [file Table1.docx]

**Supplementary Table 1:** List of antibodies used in this study

| **Primary Antibodies** | **Species** | **Producer** | **RRID** |
| --- | --- | --- | --- |
| *Anti-β-tubulin* | Mouse | Sigma-Aldrich | AB_1844090 |
| *Anti-DNAH5* | Rabbit | Sigma-Aldrich | AB_10672791 |
| *Anti-DNAH1* | Rabbit | Sigma-Aldrich | AB_10670849 |
| **Secondary Antibodies** |  |  |  |
| *Anti-Mouse IgM TRITC* | Rabbit | Sigma-Aldrich | AB_259799 |
| *Anti-Rabbit IgM FITC* | Mouse | Sigma-Aldrich | AB_1137637 |
