# Supplementary material for: Case report: The CCDC103 variant causes ultrastructural sperm axonemal defects and total sperm immotility in a professional athlete without primary ciliary diskinesia
Source: Front Genet. 2023 Jan 26;14:1062326. doi: 10.3389/fgene.2023.1062326 (PMC9908957; doi:10.3389/fgene.2023.1062326)
Supplement: Supplementary file 2 [file Table2.docx]

**Supplementary Table 2:**

| **Semen parameters** | **#1** | **#2** | **#3** | **#4** |
| --- | --- | --- | --- | --- |
| *Concentration (x 10^6^ /mL)* | 13 | 43 | 80 | 10 |
| *Total sperm count (10^6^/ejaculate)* | 26 | 645 | 120 | 30 |
| *Progressive Motility (%)* | 0 | 0 | 0 | 0 |
| *Total motility (%)* | 0 | 0 | 0 | 0 |
| *Vitality (%)* | 67 | 73 | 65 | 70 |
