# Supplementary material for: Case report: The CCDC103 variant causes ultrastructural sperm axonemal defects and total sperm immotility in a professional athlete without primary ciliary diskinesia
Source: Front Genet. 2023 Jan 26;14:1062326. doi: 10.3389/fgene.2023.1062326 (PMC9908957; doi:10.3389/fgene.2023.1062326)
Supplement: Supplementary file 3 [file Table3.docx]

**Primers used for Sanger sequencing of exon 4 of the CDCC103 gene**

FW: TTCTATCGTGATTGGCGACGA

RV: CTTAGGTTCAGGGTGAAGCG

**Results of the multiple sequence alignment of the Sanger sequencing for the proband's brother sample and the reference genome sequence (exon 4 of the gene).**

***Forward sequence alignment***


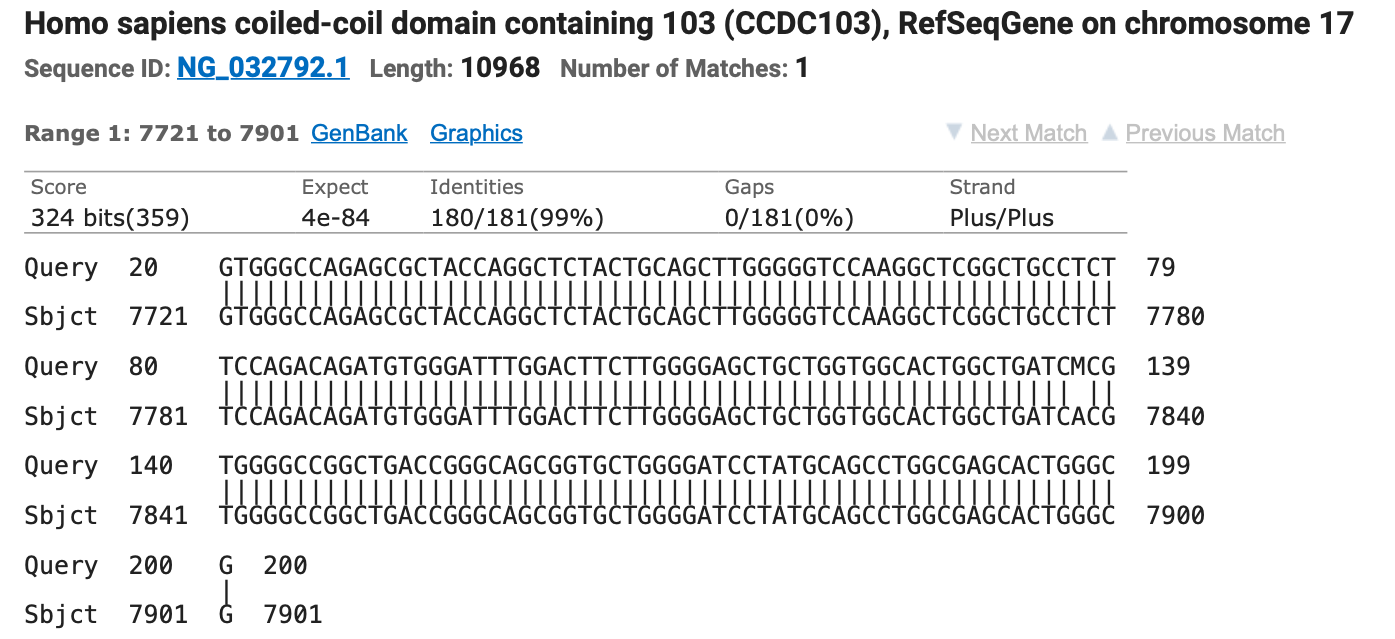


***Reverse sequence alignment***


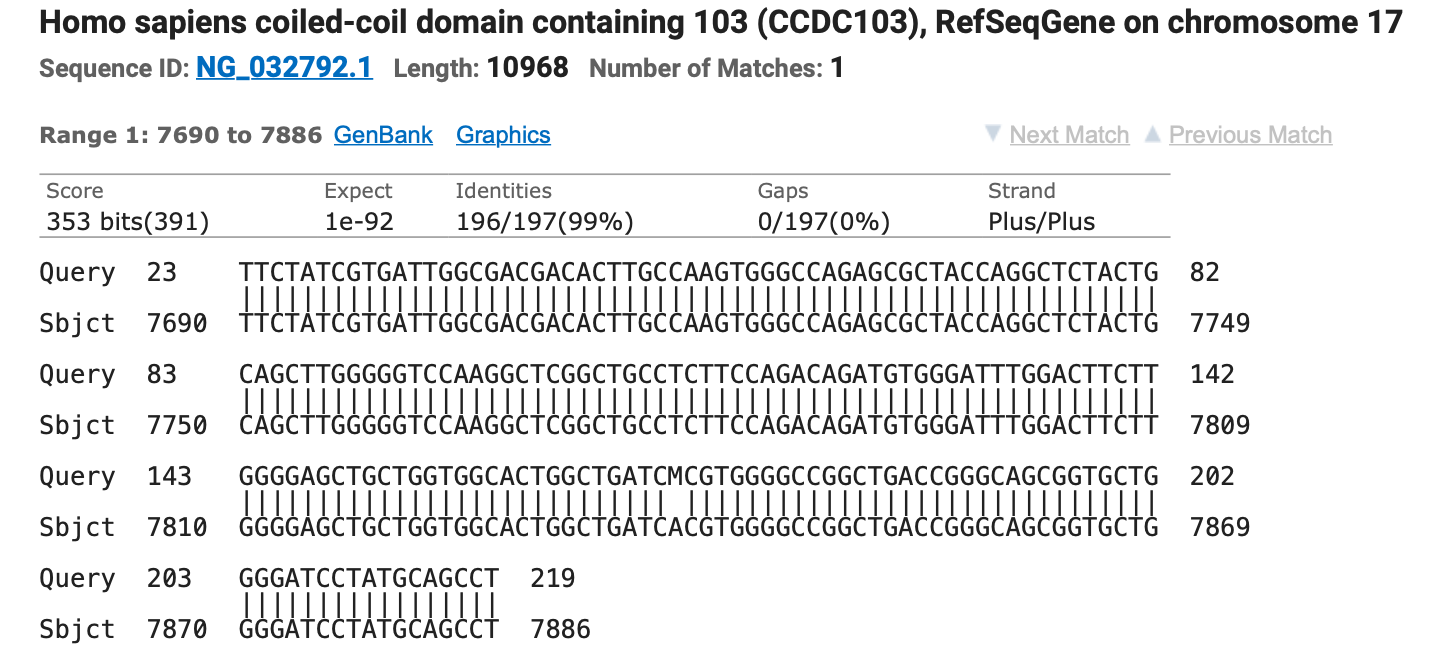


**IGV FILE**


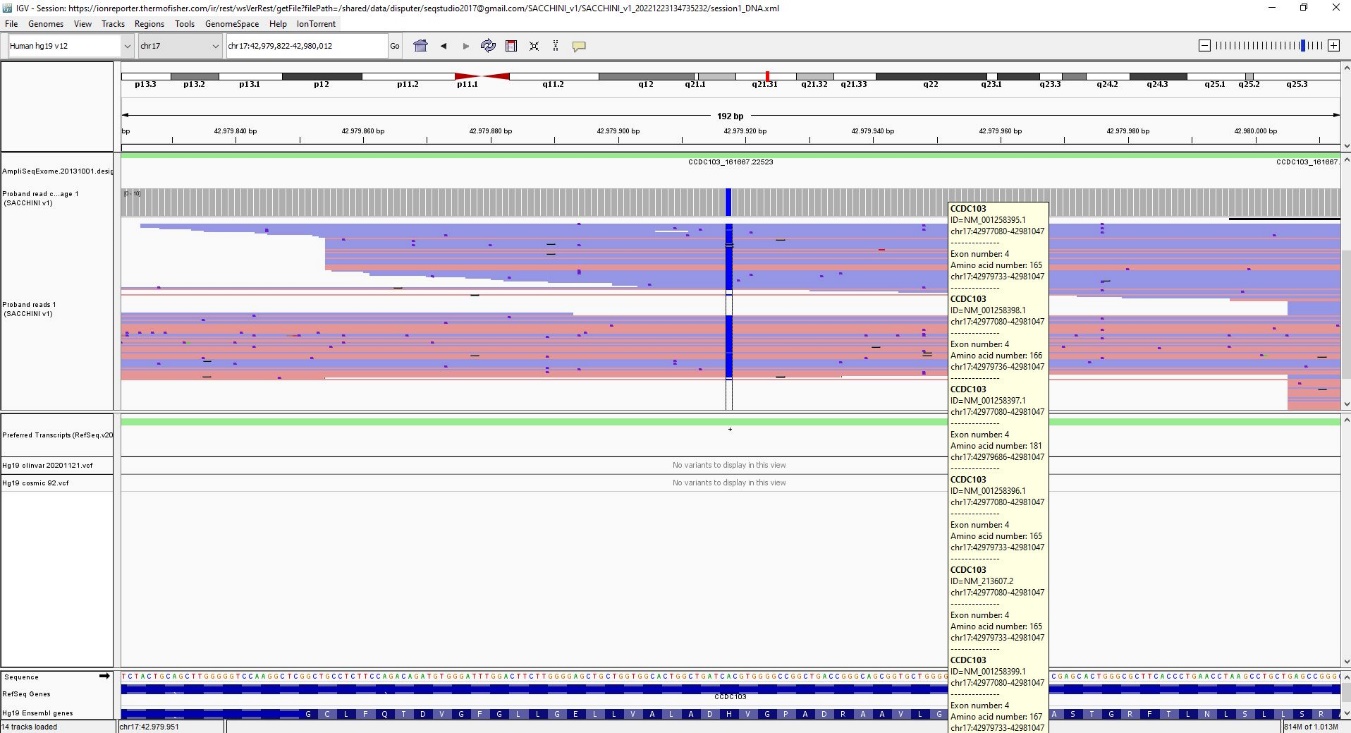


TABLE of the Genes

| GENE | OMIM | REFSEQ |
| --- | --- | --- |
| *ODAD2* | [615451](https://omim.org/entry/615451) | [NM_213607.2](https://databases.lovd.nl/shared/transcripts/00024167) |
| *CCDC39* | [613807](https://omim.org/entry/613807) | [NM_181426.1](https://databases.lovd.nl/shared/transcripts/00000266) |
| *CCDC40* | [613799](http://www.omim.org/entry/613799) | [NM_017950.3](https://databases.lovd.nl/shared/transcripts/00000267) |
| *CCDC65* | 615067 | [NM_033124.4](https://databases.lovd.nl/shared/transcripts/00004492) |
| *ODAD3* | [616037](https://omim.org/entry/615037) | NM_145045.5 |
| CCNO | 615872 | NM_021147.3 |
| CFAP298 | 615500 | NM_001350338.2 |
| DNAAF1 | 613193 | NM_178452.4 |
| DNAAF2 | 612518 | NM_001083908.1 |
| DNAAF3 | 606763 | NM_178837.4 |
| DNAAF4 | 615482 | NM_130810.3 |
| DNAAF5 | 614874 | NM_017802.4 |
| DNAAF11 | 614935 | NM_012472.4 |
| RSPH4A | 612649 | NM_001010892.2 |
| RSPH9 | 612650 | NM_152732.4 |
| SPAG1 | 615505 | NM_172218.2 |
| SPEF2 | 604418 | NM_024867.3 |
| STK36 | 607652 | NM_015690.5 |
| TTC25 | 617092 | NM_031421.2 |
| ZMYND10 | 615444 | NM_015896.2 |
| CCDC103 | 614679 | NM_001258395.2 |
| DNAH1 | 617577 | NM_024887.3 |
| DNAH5 | 608644 | NM_001369.2 |
| DNAH9 | 618300 | NM_001372.3 |
| DNAJB13 | 617091 | NM_153614.2 |
| DNAL1 | 614017 | NM_031427.3 |
| DRC1 | 615294 | NM_145038.5 |
| FOXJ1 | 613582 | NM_001454.3 |
| GAS2L2 | 618449 | NM_139285.3 |
| GAS8 | 616726 | NM_001481.2 |
| LRRC56 | 618254 | NM_198075.3 |
| PIH1D3 | 300991 | NM_001169154.1 |
| RSPH1 | 615481 | NM_080860.2 |
| *RSPH3* | 616481 | NM_031924.4 |
